# A serious nightmare: psychiatric and neurologic adverse reactions to mefloquine are serious adverse reactions

**DOI:** 10.1002/prp2.328

**Published:** 2017-06-05

**Authors:** Remington L. Nevin

**Affiliations:** ^1^ Department of Environmental Health and Engineering Johns Hopkins Bloomberg School of Public Health Baltimore Maryland

**Keywords:** Adverse effects, mefloquine, serious adverse reactions

## Abstract

Mefloquine (originally marketed as Lariam) is a neurotoxic quinoline derivative antimalarial drug that is known to cause serious and potentially lasting neuropsychiatric adverse reactions. Since 2013, drug regulators in several jurisdictions, including the United States, the United Kingdom, Ireland, and Canada, have required their mefloquine labels be updated to warn that when used for malaria prophylaxis the drug should be discontinued at the onset of neurologic or psychiatric symptoms. These recent changes to the international labeling serve to imply that psychiatric and neurologic reactions to mefloquine prophylaxis may be an early warning of an impending more serious reaction that may further jeopardize the patient with continued use of the drug. To prevent these more serious effects, these drug labels now warn that mefloquine should be discontinued and that patients seek immediate medical intervention to obtain an alternative antimalarial drug when psychiatric or neurologic symptoms occur. When used correctly for malaria prophylaxis as the updated labeling now directs, it is reasonable to expect that mefloquine will be discontinued, and an alternative drug substituted, in each patient who develops psychiatric or neurologic symptoms. This opinion discusses the implications of this updated labeling for the reporting of adverse reactions and for the continued use of the drug in malaria prophylaxis.

Mefloquine (originally marketed as Lariam) is a neurotoxic quinoline derivative antimalarial drug that is known to cause serious neuropsychiatric adverse reactions when used for malaria prophylaxis (Nevin 2012, 2014). In 2013, the U.S. Food and Drug Administration required that a boxed warning (or “black box”) be added to the mefloquine label, warning that neuropsychiatric adverse reactions could persist after mefloquine has been discontinued (Nevin and Byrd 2016). The boxed warning also directed that mefloquine prophylaxis should be discontinued and an alternative antimalarial drug substituted at the onset of psychiatric or neurologic symptoms (Nevin and Byrd 2016). Since the 2013 U.S. boxed warning, drug regulators in several jurisdictions, including the United Kingdom, Ireland (Nevin and Byrd 2016), and Canada (Nevin [Link]), have required their mefloquine labels be updated to include substantially similar warnings.

Before these changes, the U.S. label warned “if psychiatric symptoms such as acute anxiety, depression, restlessness or confusion occur, these may be considered prodromal to a more serious event” (Nevin and Byrd 2016). A little appreciated consequence of recent labeling changes is that these symptoms, as well as other psychiatric and neurologic adverse reactions to mefloquine prophylaxis that may similarly be considered prodromal should themselves arguably now be considered serious.

By standard pharmacovigilance definitions, serious adverse drug reactions include those that result in death, may be life threatening, or that result in hospitalization or significant or persistent disability or incapacity (International Conference on Harmonization 2003). By these definitions, suicide and life‐threatening adverse reactions to mefloquine, including attempted suicide (Arznei‐Telegramm 2000), are clearly serious. Psychiatric adverse reactions that require hospitalization, including psychosis (Nevin 2012), or lasting psychiatric or neurologic adverse reactions that result in disability or incapacity (Livezey et al. 2016; Nevin and Ritchie 2016) are also clearly serious.

However, serious adverse drug reactions also include other “medically important” events, such as those that might jeopardize the patient, or that might require medical intervention to prevent any of these effects (International Conference on Harmonization 2003). Arguably, adverse reactions that “may be considered prodromal to a more serious event” or that might require medical intervention to prevent death, or threats to life, or hospitalization, or significant or persistent disability or incapacity, must similarly be considered serious. Arguably, these reactions include the previously mentioned acute anxiety, depression, restlessness, and confusion. Arguably, these reactions also include other psychiatric symptoms, including nightmares or abnormal dreams, and neurologic symptoms, including symptoms of neuropathy, for which various updated labels now recommend the immediate discontinuation of mefloquine and the prescribing of an alternative antimalarial drug (Nevin and Byrd 2016).

While it is undoubtedly counterintuitive that psychiatric symptoms as seemingly mild as nightmares or abnormal dreams should be considered serious adverse reactions, mefloquine labels in the United Kingdom and Ireland now explicitly warn that psychiatric symptoms including abnormal dreams and nightmares have to be regarded as prodromal for a more serious event. These labels clearly warn “if these reactions or changes to [the patient's] mental state occur during mefloquine use, to stop taking mefloquine and seek medical advice immediately so that mefloquine can be replaced by alternative malaria medication” (Fig. 1).

**Figure 1:**
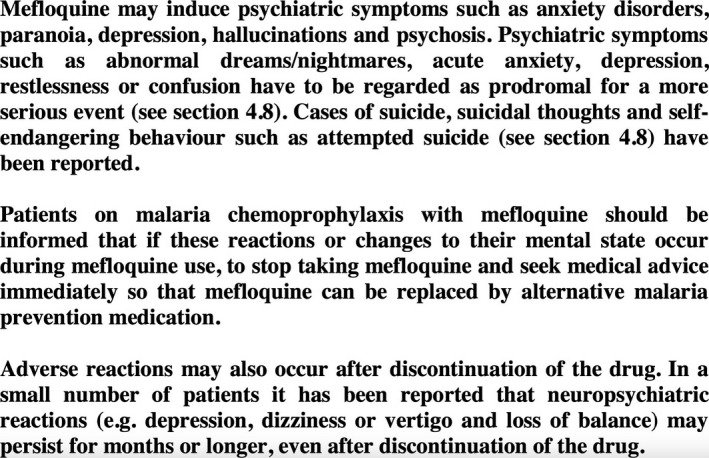
Excerpt of current U.K. mefloquine drug label (Lariam Summary of Product Characteristics, Section 4.4: Special warnings and precautions for use, dated 27 January 2017).

Similarly, the U.K. and Irish mefloquine labels now warn that mefloquine should be discontinued in patients experiencing symptoms of neuropathy, including pain, burning, tingling, numbness, and weakness, to prevent the development of an irreversible condition. Both the U.S. and Canadian mefloquine labels now warn “if neurologic symptoms occur, the drug should be discontinued and an alternative medication should be substituted” (Nevin and Byrd 2016; Nevin [Link]).

Drug regulators in Europe, on reviewing accumulated pharmacovigilance data, recently concluded there was evidence “supporting a causal relationship between mefloquine and the occurrence of long‐lasting and even persistent neuropsychiatric effects,” and speculated that these were due to “permanent brain damage.” These regulators could not identify risk factors for these effects, and concluded that “only the advice – to stop taking mefloquine if neuropsychiatric reactions or changes to their mental state occur – can be given as a precautionary measure” (European Medicines Agency, 2014).

Reflecting this conclusion, recent changes to international mefloquine labels serve to imply that psychiatric and neurologic reactions to mefloquine may indicate a patient's susceptibility to these effects and may be an early warning, or prodrome, of an impending more serious reaction that may further jeopardize the patient with continued use of the drug. To prevent these more serious effects, international mefloquine labels now warn patients to discontinue mefloquine at the onset of prodromal psychiatric and neurologic symptoms and to seek immediate medical intervention. In the context of travel to a malaria‐endemic area, such immediate medical intervention is necessary to obtain an alternative antimalarial drug to reduce the risk of this potentially fatal disease.

Mefloquine is known from double‐blinded, randomized trials to be associated with reports of neuropsychiatric effects in between 29% (Overbosch et al. 2001) and 77% (Schlagenhauf et al. 2003) of prophylactic users. Of the specific prodromal symptoms listed in the updated drug labeling for which discontinuation of mefloquine is recommended, abnormal dreams are reported in 14%, anxiety in 4%, and depression in 4% (Overbosch et al. 2001). When used correctly as the updated label now directs for malaria prophylaxis, it is reasonable to expect that mefloquine will be discontinued, and an alternative drug substituted, in each patient who develops these and other psychiatric or neurologic symptoms. Arguably, this should result in reports of serious adverse reactions from at least a sizeable minority of patients prescribed the drug, among whom the drug is expected to be discontinued.

Considering the updated international drug labeling, clinicians in these jurisdictions who continue to prescribe mefloquine should ensure that they are counseling their patients on the common incidence of symptoms for which discontinuation of the drug is recommended. These clinicians should also ensure that their patients are counseled on how to obtain an alternative antimalarial drug while traveling to a malaria‐endemic area, in the common event that such symptoms occur, and discontinuation of the drug is recommended. Finally, given the arguments presented here, these clinicians should also report symptoms including abnormal dreams and nightmares that lead to discontinuation of mefloquine to their drug regulator as serious adverse reactions. That this is generally not being done should be considered a serious nightmare for drug regulators, and for those who continue to recommend the widespread use of the drug for malaria prophylaxis.

## Disclosure

I have been retained as consultant and expert witness in legal cases involving claims of adverse effects from antimalarial drugs.
